# Treatment with Ataluren for Duchene Muscular Dystrophy

**DOI:** 10.15844/pedneurbriefs-34-12

**Published:** 2020-12-04

**Authors:** Sameh S. Morkous

**Affiliations:** 1 Department of Pediatrics, Pediatric Neurology Division, Lehigh Valley Children's Hospital, Allentown, PA

**Keywords:** Pediatric, Neuromuscular, Duchenne Muscular Dystrophy, STRIDE Registry

## Abstract

Investigators from Europe and the USA, representing the STRIDE Registry and Cooperative International Neuromuscular Research Group (CINRG) Duchenne Natural History Study (DNHS), examined the effectiveness of ataluren and standard of care in the Registry versus stand of care alone in the CINRG DNHS.

Investigators from Europe and the USA, representing the STRIDE Registry and Cooperative International Neuromuscular Research Group (CINRG) Duchenne Natural History Study (DNHS), examined the effectiveness of ataluren and standard of care in the Registry versus stand of care alone in the CINRG DNHS. The CINRG DNHS was a prospective, longitudinal worldwide study of more than 400 patients with Duchene Muscular Dystrophy (DMD) followed between 2006 and 2016. This analysis indicated that ataluren and standard of care delays DMD progression of functional milestones in patients with nmDMD and that ataluren was well tolerated. [1]

COMMENTARY. The clinical potential of ataluren in the treatment of DMD was described by Namgoong et al. [2]. Ataluren is a first-in-class, oral treatment for patients with nmDMD, designed to enable full-length dystrophin protein production. Ataluren has been evaluated previously in patients with nmDMD in two randomized controlled trials. Both trials showed that ataluren (40 mg/kg/day) had favorable functional efficacy. Ataluren is indicated for the treatment of nmDMD in ambulatory patients aged five years or older in Brazil, Chile, Israel, the Republic of Korea, Ukraine, and two years or older in Iceland, Liechtenstein, and Norway. Efficacy has not been demonstrated in non-ambulatory patients.

The STRIDE Registry constitutes the first drug registry for patients with DMD. Mean & Standard deviation (SD) ages of patients at muscle biopsy and genetic diagnosis were 4.5 (2.5) years and 5.2 (2.9) years, respectively; the time from first symptoms to genetic diagnosis was 2.4 (2.4) years. Results from a separate international multicenter registry study showed that the mean (SD) patient age at DMD diagnosis by muscle biopsy or genetic testing was 4.3 (2.5) years, and the mean (SD) time from first symptoms to this diagnosis was 1.3 (1.8) years across countries. These figures suggest that patients in the STRIDE Registry are diagnosed later than those in the total DMD population. This phenomenon is probably related to the sequential genetic testing process for DMD introducing delays in diagnosis. However, compared with five years ago, next-generation sequencing is now more accessible and less expensive; thus, performing the second step in the genetic testing process is more feasible now, closing this diagnostic delay [3].

The STRIDE Registry provided the opportunity to follow-up patients over a more extended period than clinical studies. The study's limitation is that the STRIDE and CINRG DNHS populations were not matched according to nmDMD mutation type or location. However, this would not be considered a real source of bias because patients were matched based on other factors that predict disease progression, such as age at onset of first symptoms (4). Overall, the results corroborate previous evidence that ataluren treatment can slow disease progression in nmDMD. The STRIDE Registry contains patients with a broader range of ages and ambulatory ability than those in clinical trials, and thus, the data represents a broader range of real-world experiences [3].

These analyses are based on interim data, but the STRIDE Registry study's final results are expected 2025. Large clinical trials are required to assess ataluren's role and its long-term impact on disease progression in non-ambulant nmDMD patients, but the introduction of ataluren in the field is an achievement [2].

## Disclosures

The author has declared that no competing interests exist.
